# First Characterization of the* Neospora caninum* Dense Granule Protein GRA9

**DOI:** 10.1155/2017/6746437

**Published:** 2017-11-12

**Authors:** Margret Leineweber, Katrin Spekker-Bosker, Vanessa Ince, Gereon Schares, Andrew Hemphill, Silvia K. Eller, Walter Däubener

**Affiliations:** ^1^Institute of Medical Microbiology and Hospital Hygiene, Heinrich-Heine-University, Düsseldorf, Germany; ^2^Federal Research Institute for Animal Health, Institute of Epidemiology, Friedrich-Loeffler-Institute, Greifswald-Insel Riems, Germany; ^3^Institute of Parasitology, Vetsuisse Faculty, University of Berne, Berne, Switzerland

## Abstract

The obligate intracellular apicomplexan parasite* Neospora caninum (N. caninum)* is closely related to* Toxoplasma gondii (T. gondii)*. The dense granules, which are present in all apicomplexan parasites, are important secretory organelles. Dense granule (GRA) proteins are released into the parasitophorous vacuole (PV) following host cell invasion and are known to play important roles in the maintenance of the host-parasite relationship and in the acquisition of nutrients. Here, we provide a detailed characterization of the* N. caninum* dense granule protein NcGRA9. The in silico genomic organization and key protein characteristics are described. Immunofluorescence-based localization studies revealed that NcGRA9 is located in the dense granules and is released into the interior of the PV following host cell invasion. Immunogold-electron microscopy confirmed the dense granule localization and showed that NcGRA9 is associated with the intravacuolar network. In addition, NcGRA9 is found in the “excreted secreted antigen” (ESA) fraction of* N. caninum*. Furthermore, by analysing the distribution of truncated versions of NcGRA9, we provide evidence that the C-terminal region of this protein is essential for the targeting of NcGRA9 into the dense granules of* N. caninum*, and the truncated proteins show reduced secretion.

## 1. Introduction

*Neospora caninum (N. caninum)* is an obligate intracellular parasite and a member of the phylum Apicomplexa.* N. caninum* is closely related to* Toxoplasma gondii (T. gondii)*, and both undergo a heteroxenous life cycle [1]. The definitive hosts of* N. caninum* are members of the canid family [2]. Although a wide range of wild and domestic animals get into contact with* N. caninum,* viable parasites could be isolated from only few intermediate hosts [3]. The economically most important intermediate host of* N. caninum* is cattle, and infection takes place by the oral uptake of oocyst-contaminated food, water, or soil.* N. caninum* can persist as tissue cyst-forming stages, bradyzoites, mainly in skeletal muscles or neural tissues. Most importantly, pregnancy can cause recrudescence and* N. caninum* tachyzoites can be transmitted vertically from the dam to the fetus, and such a transmission often leads to abortion or to asymptomatic or symptomatic chronic infections in the offspring [1].

Similar to most other apicomplexan parasites,* N. caninum* exhibits three morphologically distinct secretory organelles named micronemes, rhoptries, and dense granules [4]. The content of these organelles is coordinately secreted during the parasitic invasion process, ensuring proper adhesion to the host cell surface membrane, reorientation, and host cell entry, as well as survival and further development in the parasitophorous vacuole (PV) [5]. Dense granule (GRA) proteins are secreted, at high levels shortly after host cell invasion and then constitutively at a lower level thereafter. These proteins have been proposed to be involved in host-parasite relationship and in nutrient acquisition. In* N. caninum*, several GRA proteins were described, which exhibit homology to respective* T. gondii* proteins. Until now, no precise function for any of the GRA proteins has been described in* N. caninum*. In addition, the majority of the GRA proteins do not show any significant similarity to known proteins except to other GRA proteins in apicomplexan parasites [6].

Here we describe in detail the* N. caninum* GRA protein NcGRA9 whose existence was postulated previously [7, 8]. A putative GRA9 protein had been identified in the lysates of* N. caninum* bradyzoites and in the secreted fraction of tachyzoites using mass spectrometry (MS), respectively. In this study, we analysed NcGRA9 on the genomic and on the protein level. NcGRA9 exhibits a high sequence homology to TgGRA9 and contains a typical N-terminal signal peptide and an intracellular localization pattern. In addition, we identified the importance of the C-terminal region of NcGRA9 for the proper targeting of the protein to the dense granules using two C-terminally truncated NcGRA9 variants.

## 2. Methods

### 2.1. Cell Culture

Human foreskin fibroblasts (HFF, ATCC, Wesel, Germany) were cultivated in Iscove's Modified Dulbecco's Medium (IMDM, Lonza, Rockland, USA) supplemented with 10% FCS (Lonza, Rockland, USA; heat-inactivated at 56°C for 30 minutes).* Neospora caninum* (Nc-1 strain, provided by Friedrich Loeffler-Institute, Greifswald-Insel Riems, Germany) was grown on confluent monolayers of HFF and parasites were transferred to new feeder cells twice a week.

### 2.2. Harvesting of Extracellular* N. caninum* Tachyzoites from Cell Cultures

Extracellular* N. caninum* tachyzoites were harvested by differential centrifugation: first at 100 ×g (10 min) and then the supernatant at 2500 ×g (10 min). Viable parasites were counted after Trypan blue (Sigma-Aldrich, St. Louis, USA) staining.

### 2.3. Isolation of Genomic DNA

For isolation of genomic* N. caninum* DNA, tachyzoites were grown in 162 cm^2^ cell culture flasks (cell culture treated, 0.2 *μ*m ventilation cap, costar 3151, Corning Inc., Corning, USA) and harvested. 1 × 10^8^ tachyzoites were resuspended in Laird's tail buffer (100 mM Tris/HCl (pH 8.5), 5 mM EDTA (pH 8), 0.2% SDS, 200 mM NaCl). After addition of 0.2 mg Proteinase K (Roche, Mannheim, Germany), the sample was incubated at 55°C overnight. Genomic DNA was isolated and precipitated using phenol/chloroform extraction and solubilized in water, and the concentration was determined by a NanoDrop 1000 Spectrophotometer (Thermo Scientific, Waltham, Massachusetts, USA). Genomic DNA was stored at −20°C.

### 2.4. RNA Isolation and cDNA Synthesis

1 × 10^8^ tachyzoites were harvested as described above and the RNA was isolated similar to the single-step-method by Chomczynski and Sacchi [9] by addition of Tri Reagent (Sigma-Aldrich, St. Louis, USA). In brief, tachyzoites were resuspended in Tri Reagent. After a short incubation at RT, chloroform (Sigma-Aldrich, St. Louis, USA) was added and after short incubation the sample was centrifuged (17000 ×g, 15 min, 4°C). For RNA precipitation the upper aqueous phase was transferred to a new tube and mixed with isopropanol (Sigma-Aldrich, St. Louis, USA). After a short incubation at RT the sample was centrifuged (17000 ×g, 10 min, 4°C), the RNA pellet was washed with 75% ethanol, air-dried, and dissolved in DEPC water. The RNA concentration was determined by a NanoDrop 1000 Spectrophotometer and RNA was stored at −80°C.

For cDNA synthesis, 1 *μ*g RNA was diluted in DEPC water, and cDNA was synthesized using M-MLV Reverse Transcriptase (life technologies, Carlsbad, USA) according to the manufacturer's instructions with some modifications: 20 U RNase OUT Ribonuclease Inhibitor, incubation for 1 h at 37°C followed by 4 min at 95°C. After addition of 80 *μ*l DEPC water cDNA concentration was measured by a NanoDrop 1000 Spectrophotometer and stored at −20°C.

### 2.5. PCR Analyses of the Exon-Intron Structure of NcGRA9

For PCR analyses two different primer pairs were used (1-1697for and 1-1697rev; 271-1290for and 271-1290rev) (Table 1). The PCR reaction mix was prepared in a total volume of 50 *μ*l, and the Expand High Fidelity PCR System (Roche, Mannheim, Germany) was employed. 100 ng genomic DNA or cDNA and 50 pmol per primer were used. Thermal cycling was carried out in a thermocycler PTC-200 (MJ Research, Bio-Rad Laboratories, Hercules, USA) using the following settings: 3 min 94°C; 30 times: 30 sec 94°C, 30 sec 57°C, 70 sec 72°C; 7 min 72°C; ∞ 4°C. PCR products were loaded on 1% agarose gel (Lonza, Rockland, USA) and the gel was documented by Gel Doc XR^+^ (Biorad, Hercules, USA).

### 2.6. Quantitative Real-Time PCR

1 × 10^4^ HFF were seeded in 96-well plates and incubated over night at 37°C. After 24 h, cells were infected with 1 × 10^5^* N. caninum* for 72 h in triplicate.

For DNA isolation the 96-well plates were frozen at −20°C to lyse the cells and parasites. The samples were resuspended and the triplicates were pooled. The samples were centrifuged at 17000 ×g (30 min), supernatants were removed and pellets were resuspended in 25 *μ*l PBS. 50 *μ*l Proteinase K buffer (100 *μ*g/ml Proteinase K in minimal TE-buffer (1 M Tris/HCl (pH 8), 0.5 M EDTA)) was added followed by incubation for 60 min 56°C and 30 min 96°C. The samples were diluted 1 : 100 in TE-buffer. PCR reaction mix to amplify Nc5 region of* N. caninum*: 5 *μ*l diluted sample, 12.5 *μ*l qPCR® Mastermix-No ROX (Eurogentec, Seraing, Belgien), 7.5 pmol per primer (Nc5forqPCR, Nc5revqPCR (Table 1)), 5 pmol probe and H_2_O @ 25 *μ*l. Amplification protocol was as follows: cycle 1 step 1: 2 min 50°C, step 2: 10 min 95°C; cycle 2 (45 times) step 1: 15 sec 95°C, step 2: 1 min 60°C; cycle 3: ∞ 4°C. The results were analysed by iQ5 software (Bio-Rad Laboratories, Hercules, USA). DNA of defined numbers of tachyzoites was used as standard (10^5^, 10^4^, 10^3^, 10^2^, 10^1^).

### 2.7. Southern Blot Analysis

The probe was amplified by PCR on* N. caninum* DNA, using the primers probeNcGRA9for and probeNcGRA9rev (Table 1). After gel electrophoresis DNA was purified employing the Zymoclean Gel DNA Recovery Kit (Zymoresearch, Irvine, USA) and the purified PCR product was ligated into the TOPO TA vector system (pCR2.1 Life Technologies, Carlsbad, USA) according to manufacturer's instructions.

After transformation of* Escherichia coli* and culture of transformants (Luria-Bertani (LB) broth, 37°C, shaking), plasmid DNA was purified using the QIAGEN Plasmid Midi or Maxi Kit (Qiagen, Hilden, Germany) according to manufacturer's instructions. Plasmids were digested with* EcoR*I (Fermentas, Burlington, USA) according to manufacturer's instructions, separated by agarose gel electrophoresis, and the probe was purified as described above.

For Southern Blot analysis, 40 *μ*g genomic DNA was digested with* Hind*III,* BamH*I,* Nde*I, and* Sma*I (New England Biolabs, Ipswich, USA), respectively, according to manufacturer's instructions. After DNA separation by gel electrophoresis, the gel was incubated for 15 min in 0.25 N HCl followed by 30 min incubation in 0.4 M NaOH. DNA was blotted by alkaline blot on a positive charged Nylon membrane (Hybond N^+^, Amersham, GE Healthcare, Buckinghamshire, UK) over night. The membrane was washed for 15 min in 2x SSC (20x SSC: 0.3 M sodium citrate, 3 M NaCl, pH 7.0; diluted in water), air-dried, and incubated for 30 min at 80°C. For hybridization, the membrane was moistened with 2x SSC and prehybridized for 1 h at 60°C in ExpressHyb™ Hybridization Solution (Clontech Laboratories, Mountain View, USA). 25 ng probe was used for hybridization which was carried out at 60°C overnight with Ladderman™ Labeling Kit (Takara, Shiga, Japan) according to manufacturer's instructions. Thereafter, the membrane was washed with 2x SSC/0.05% SDS at RT and with 0.1x SSC/0.1% SDS at 50°C. For analysis a radiographic film (Kodak film BioMax MR, Rochester, USA) was exposed at −80°C.

### 2.8. Expression of Recombinant NcGRA9 in* E. coli* and Production of a Polyclonal Anti-NcGRA9 Antiserum

For production of recombinant protein, the cDNA sequence coding for NcGRA9 was amplified by PCR (primers: NcGRA9NdeIfor, NcGRA9HindIIIre) (Table 1). The expression vector pET22b(+) (Novagen, Darmstadt, Germany) was digested with* Nde*I and* Hind*III (Fermentas, Ipswich, USA) and ligated with the PCR product according to manufacturer's instructions (T4 ligase, Life Technologies, Carlsbad, USA). After transformation of* E. coli* expression strain BL-21 by heat shock, bacterial cultures were grown to OD_600 nm_ 0.3 in 500 ml LB broth with ampicillin. Protein expression was induced by adding 2 mM IPTG (Isopropyl *β*-D-1-thiogalactopyranoside, Fermentas, Ipswich, USA);* E. coli* were incubated for 2 h at 37°C and were subsequently centrifuged at 11300 ×g (15 min, 4°C, J2-21 Centrifuge, Beckman, Brea, USA). The pellet was resuspended in a denaturing buffer A (6 M guanidine hydrochloride, 0.1 M NaH_2_PO_4_, 0.01 M Tris/HCl, pH 8), frozen in liquid nitrogen, and thawed at 37°C in a water bath. The sample was incubated for 30 min (RT) on a rotating wheel and centrifuged at 17000 ×g (15 min). Meanwhile, 600 *μ*l Ni-NTA agarose (QIAGEN, Hilden, Germany) was twice equilibrated in buffer A. Equilibrated Ni-NTA was mixed with bacteria lysate and the sample was incubated for 60 min (RT) on a rotating wheel. The lysate-Ni-NTA suspension was loaded onto a disposable column (QIAGEN, Hilden, Germany) and washed with 10 ml of the following buffers: buffer A, buffer B (8 M Urea, 0.1 M NaH_2_PO_4_, 0.01 M Tris/HCl, pH 8), and buffer C (8 M Urea, 0.1 M NaH_2_PO_4_, 0.01 M Tris/HCl, pH 6.3). The protein was eluted from the Ni-NTA resin with 500 *μ*l buffer E (8 M Urea, 0.1 M NaH_2_PO_4_, 0.01 M Tris/HCl, 0.25 M Imidazol, pH 6.3).

For immunization of two rabbits, purified recombinant NcGRA9 was sent to Eurogentec (Seraing, Belgium) in order to raise a polyclonal protein antiserum (87-day classic polyclonal antibody protocol: first immunization and three boost immunizations with 100 *μ*g purified recombinant NcGRA9 each).

### 2.9. Cloning of NcGRA9-HA Variants into pDHFR-TSc3 and Stable Transfection of* N. caninum*

The vector system pDHFR-TSc3 [10] was utilized to transfect* N. caninum*. After digestion with* Spe*I and* Not*I (all restriction endonucleases New England Biolabs, according to manufacturer's instructions) the promoter region of DHFR was inserted (primers for amplification: pDHFRSpeINotIfo, pDHFRSpeINotIre). After digestion with* Avr*II and* Nco*I NcGRA9-HA was inserted (amplification primers: GRA9HAAvrNcofor, GRA9HAAvrNcorev). After digestion with* Nco*I and* Not*I 3′ UTR (337 bp) of NcGRA9 was inserted into the vector (amplification primers: 3′GRA9NcoINotIf, 3′GRA9NcoINotIr). To insert the C-terminal truncations of NcGRA9 the vector was digested with* Avr*II and* Nco*I (reverse primers: NcGRA9d280revHA, NcGRA9d201revHA) (Table 1).

To stably transfect* N. caninum* with pDHFR-TSc3-full-lengthNcGRA9-HA, pDHFR-TSc3-Δ280NcGRA9-HA, and pDHFR-TSc3-Δ201NcGRA9-HA freshly liberated parasites were harvested and washed in PBS by centrifugation (2500 ×g). The tachyzoites were resuspended in 800 *μ*l Cytomix (120 mM KCl, 0.15 mM CaCl_2_, 10 mM K_2_HPO_4_/KH_2_PO_4_, 25 mM Hepes, 2 mM EGTA, 5 mM MgCl_2_, pH 7.6). After addition of 3 mM ATP and 3 mM glutathione the parasites were incubated for 5 min (RT). After addition of 100 *μ*g plasmid-DNA parasites were electroporated in Gene Pulser® Cuvette (0.4 cm electrode, Bio-Rad Laboratories, Hercules, USA) at 50 Ω and 2 kV.* N. caninum* was cultivated on HFF under pyrimethamine selection pressure (final concentration 1 *μ*M).

### 2.10. *In Silico* Analyses

The analyses of DNA and protein sequences were performed with Lasergene (DNAStar, Madison, USA). For all sequencing and cloning purposes, Clone Manager 9 Professional Edition (Scientific & Educational Software, Cary, USA) was used.

### 2.11. Preparation of* N. caninum* Lysate Antigen (NLA)

Extracellular tachyzoites were harvested as described above and after washing with PBS the tachyzoites were resuspended in water supplemented with 1x COMPLETE protease inhibitor cocktail (Roche, Mannheim, Germany; in the following referred to as protease inhibitor cocktail). The parasites were lysed by ten freeze-thaw cycles in liquid nitrogen, insoluble debris was removed by centrifugation at 1200 ×g (15 min), and the supernatant was collected and stored at −20°C.

For the preparation of lysates of* N. caninum* infected cultures, the medium was removed at 24 h postinfection and infected monolayers were washed with PBS. After addition of protease inhibitor cocktail, cells were collected with a cell scraper, passed through a 25-gauge needle three times, and the sample was centrifuged at 36 ×g (10 min). After five freeze-thaw cycles, the sample was centrifuged at 1200 ×g (10 min) to remove parasite debris, and the supernatant was stored at −20°C.

### 2.12. Preparation of Excreted Secreted Antigens (ESA) from* N. caninum*

1 × 10^8^ tachyzoites/ml were resuspended in cold medium with 10% FCS followed by incubation for different time points at 37°C under mild agitation. The parasites were centrifuged at 3600 ×g (10 min, 4°C), the supernatant was filtered through a 0.22 *μ*m membrane, 1x protease inhibitor cocktail was added, and samples were stored at −20°C.

### 2.13. Immunofluorescence Analysis

Confluent HFF were grown on glass coverslips in 24-well plates and were infected with* N. caninum* tachyzoites, either NC-1 isolate or different clones expressing variants of hem-agglutinin-tagged NcGRA9 (NcGRA9-HA variants). Infected cultures were maintained for different time spans, depending on the experiments as indicated in the text. The immunofluorescent staining was performed according to Guinoaud et al. (2010) with the following adaptations: methanol/acetone permeabilization for 20 min, final DAPI concentration 0.5 *μ*g/ml, coverslips mounted in Fluoromount-G (Southern Biotech, Birmingham, USA), and analyses with Zeiss LSM 780 confocal microscope.

For immunolabeling of extracellular* N. caninum* tachyzoites the parasites were harvested as described above, suspended in medium and small droplets of the suspension was placed onto coverslips. The samples were air-dried and immunofluorescence staining was performed as described above.

Primary antibodies used were rabbit anti-NcGRA9 antiserum or rabbit anti-HA (Invitrogen, Carlsbad, USA), both used at a dilution of 1 : 1000. As secondary antibodies goat anti-rabbit cy2 or cy3 (Dianova, West Grove, USA) were used at 1 : 1000 dilution.

### 2.14. Immunogold-Labeling and Transmission Electron Microscopy

Human foreskin fibroblast cultures infected with* N. caninum* tachyzoites were grown in T25 culture flasks and fixed and processed for embedding in LR-White resin as described (Guinoaud et al. 2010). Sections were cut on a Reichert & Jung ultramicrotome and placed onto carbon-formvar coated nickel grids (Plano GmbH, Marburg, Germany). Nonspecific binding sites were blocked for 2 h in PBS/1% BSA. Specimens were then incubated in anti-NcGRA9 antiserum diluted 1 : 500 in PBS/0.1% BSA for 1 h. After washing in 5 changes of PBS (2 min each), the goat anti-rabbit antibody conjugated to 10 nm diameter gold particles (Aurion, Wageningen, Netherlands) was applied at a dilution of 1 : 5 in PBS/0.1% BSA. After extensive washing in PBS, grids were air-dried and stained with lead citrate and uranyl acetate. Specimens were viewed on a Philips 400 TEM (Philips Electronics, Eindhoven, Netherlands) operating at 80 kV.

### 2.15. Cell Fractionation by Ultracentrifugation

For biochemical fractionation of infected HFF by ultracentrifugation, HFF (cultivated in 150 cm^2^ cell culture flasks) infected with 1 × 10^8^* N. caninum* for 24 h were used. To harvest the intracellular tachyzoites medium was discarded, the cell layer was washed with PBS, harvested with a cell scraper in 1 ml PBS/protease inhibitor cocktail/1 mM EGTA, and forced through a 25-gauge needle three times. Further treatments including ultracentrifugation were performed as described previously [11]. For Western Blot analyses, 5% of each the low speed supernatant (LSS) and high speed supernatant (HSS) were precipitated with 10% TCA (final concentration) and 5% of the respective pellets (low speed pellet (LSP) and high speed pellet (HSP)) were resuspended in PBS.

### 2.16. Western Blot Analysis

For Western Blot analysis, samples were mixed with 5x SDS sample buffer (10% SDS, 0.4 M Tris/HCl (pH 6.8), 25% glycerol, 5%  *β*-mercaptoethanol, 0.1% bromophenol blue), boiled at 100°C for 5 min and loaded on NuPAGE 10% Bis-Tris SDS gels (Life Technologies, Carlsbad, USA). Proteins were blotted on PROTRAN Nitrocellulose Transfer Membrane (Whatman, Dassel, Germany) by semidry blotting. The membranes were blocked with 5% skim milk powder (Oxoid Microbiology Products, Hampshire, England) in PBS (1 h, RT); the primary antibodies were diluted in 0.5% skim milk/PBS (antiserum anti-NcGRA9 1 : 1000, monoclonal anti-NcSAG1 antibody 1 : 2000 [12], monoclonal anti-NcGRA7 (Mab 4.11.5; [13]) antibody 1 : 100, rabbit anti-HA antibody 1 : 1000 (Life Technologies, Carlsbad, USA); 1 h RT). The membranes were washed three times in 0.2% Tween 20 (Merck, Darmstadt, Germany)/PBS for 5 min. The secondary peroxidase-conjugated antibodies (goat anti-mouse, goat anti-rabbit, Jackson ImmunoResearch Laboratories, West Grove, USA) were diluted 1 : 5000 in 0.5% skim milk/PBS and applied for 45 min at RT. After washing, the membranes were incubated in ECL Western Blotting Detection Reagents (Amersham, GE Healthcare, Buckinghamshire, UK) and exposed to Hyperfilm ECL (Amersham, GE Healthcare, Buckinghamshire, UK).

For analyses of sera from* N. caninum* infected cattle, nitrocellulose membranes were blocked in 5% horse serum/PBS, and sera and antibodies were diluted in 0.5% horse serum/PBS. Signal detection of secondary alkaline phosphatase-conjugated anti-bovine antibodies (1 : 5000; Dianova, West Grove, USA) was carried out with NBT (Sigma-Aldrich, St. Louis, USA) and BCIP-T (Fermentas, Ipswich, USA) in alkaline phosphatase buffer (100 mM Tris/HCl (pH 9.5), 100 mM NaCl, 10 mM MgCl_2_).

Some of the tested sera from* N. caninum* infected cattle were previously described. Serum 1 was obtained from a calf that was 17 days old and had* N. caninum* specific IFAT titer of 1 : 800 [14]. Further, serum 2 was obtained by cattle that was > one year old and* N. caninum* positive in immunoblot. Sera 3 and 4 were from calves that were < 0.5 months old and had* N. caninum* specific IFAT titers of 1 : 1000 and 1 : 200 [15].

### 2.17. Data Analyses and Statistical Tests

All experiments were done in triplicate and data are given as mean +/− standard error of the mean of three independent experiments. For statistical analysis the one-sample *t*-test was used. The analysis was performed with GraphPad Prism 5 software (GraphPad Software Inc., San Diego, CA).

## 3. Results

### 3.1. Molecular Characterization of the* Ncgra9* Gene and Predicted Protein Sequence

To identify the sequence of the putative homologue NcGRA9, the TgGRA9 cDNA sequence was analysed by BLAST (Basic Local Alignment Search Tool, discontiguous megablast) on the NCBI (National Center for Biotechnology Information) database. One homologous sequence was discovered in the* N. caninum* genome, designated NCLIV_066630 (data not shown). This sequence is located on chromosome XII (nucleotide 5,079,007 to 5,080,703) and the genomic sequence, coding sequence, predicted RNA/mRNA sequence, and predicted protein sequence were determined on the predicted protein database (ToxoDB Toxoplasma Genomics Resource). The coding sequence was confirmed by sequencing (data not shown).

**Figure 1:**
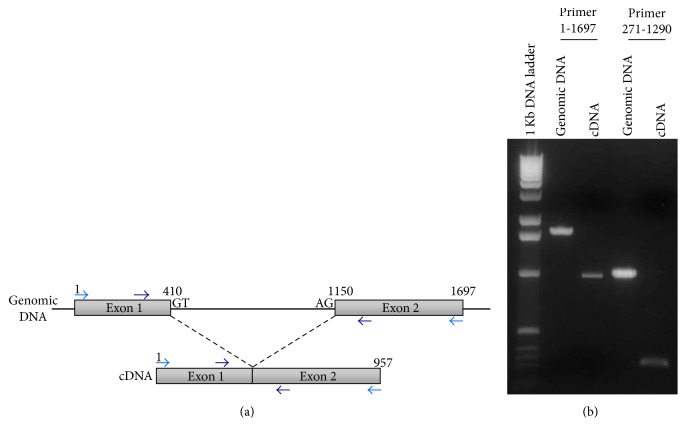
(a) In silico analysis reveals that the genomic sequence of NcGRA9 consists of one intron and two exons. The scheme depicts the genomic organization and the cDNA structure of NcGRA9. (b) The existence of the intron was confirmed by PCR on genomic DNA and cDNA with two different primer pairs (pp1 and pp2). The PCR products had the expected size by amplification with pp1 of 1697 bp (genomic DNA) or 957 bp (cDNA) and with pp2 of 1019 bp (genomic DNA) or 278 bp (cDNA).

The theoretical exon-intron structure of* Ncgra9* published on ToxoDB was verified by PCR with two different primer pairs (Figures 1(a) and 1(b)). Primer pair 1 (pp1: 1-1697for, 1-1697rev) covers the whole genomic sequence and primer pair 2 (pp2: 271-1290for, 271-1290rev) covers the middle part of the sequence spanning the intron. PCR was performed on genomic DNA and on cDNA of* N. caninum*. PCR products amplified on cDNA (pp1: 957 bp, pp2: 278 bp) were smaller than the fragments amplified on genomic DNA (pp1: 1697 bp, pp2: 1019 bp) and showed the expected sizes calculated from the published sequences. The gene structure was further characterized by the presence of splice donor (GT) and splice acceptor (AG) sites in the intron region of NcGRA9 as indicated in Figure 1(a). These results demonstrate that indeed one intron is included in the genomic* Ncgra9* sequence as it was suggested on ToxoDB.

**Figure 2:**
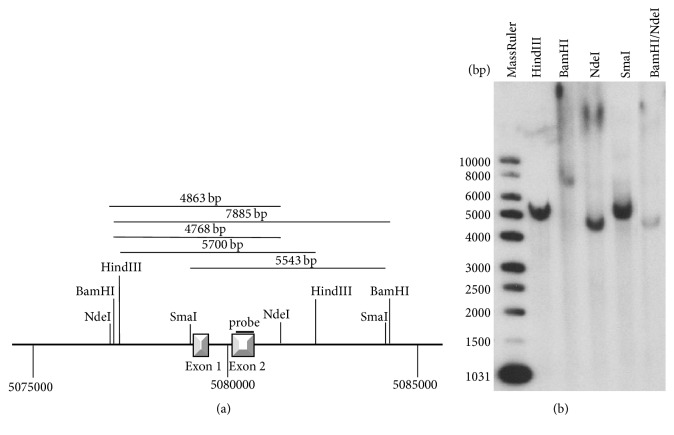
NcGRA9 is a single-copy gene in the haploid tachyzoite genome of* N. caninum*. (a) depicts a schematic representation of the genomic region flanking* Ncgra9* on chromosome XII. Restriction endonuclease recognition sites which are not placed in the exons are indicated. The probe used for Southern Blotting recognizes a sequence in the second exon. (b) shows Southern Blot analyses after enzymatic restriction of genomic DNA with different restriction endonucleases. In each case the probe detects a single band which implies that NcGRA9 is a single-copy gene in the haploid tachyzoite genome of* N. caninum*.

*N. caninum* tachyzoites have a haploid genome. By Southern Blot analysis, we examined whether* Ncgra9* is a single-copy gene. The hybridization probe was designed to bind a sequence in the second exon of* Ncgra9* as indicated in Figure 2(a). The Southern Blot shown in Figure 2(b) illustrates that after digestion with all restriction enzymes only a single band was detectable.

**Figure 3:**
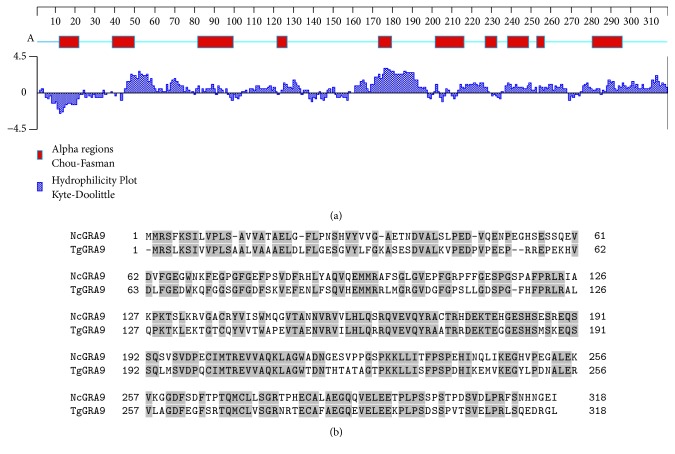
In (a), an in silico analysis of the NcGRA9 protein sequence according to Chou and Fasman [17] is shown which results in the identification of several alpha helical regions. Furthermore, the hydrophilicity of NcGRA9 was analysed according to Kyte and Doolittle [16]. This analysis characterizes NcGRA9 as a mainly hydrophilic protein. At the N-terminus a hydrophobic sequence is found, consistent with the predicted signal peptide. In (b) an alignment of NcGRA9 and TgGRA9 is depicted. The proteins have a sequence identity of about 60%. Identical amino acids are marked by grey boxes.

The predicted NcGRA9 protein sequence was further analysed in silico. The Kyte and Doolittle prediction tool [16] showed that NcGRA9 is a mainly hydrophilic protein (Figure 3(a)). Analysis by Signal P4.1 (http://www.cbs.dtu.dk/services/SignalP/) postulated a putative hydrophobic signal peptide at the N-terminus, with a predicted cleavage site between amino acids 19 and 20. According to Chou and Fasman [17] the NcGRA9 secondary structure is composed of several alpha helical regions (Figure 3(a)).

Protein sequences of TgGRA9 and NcGRA9 were retrieved from the databases and were aligned, revealing amino acid sequence identity of 60% (Figure 3(b)) and a homology of 73% (as determined by number of residues that are identical or have similar chemical properties in BLAST).

### 3.2. Localization and Biochemical Characterization of the NcGRA9 Protein

**Figure 4:**
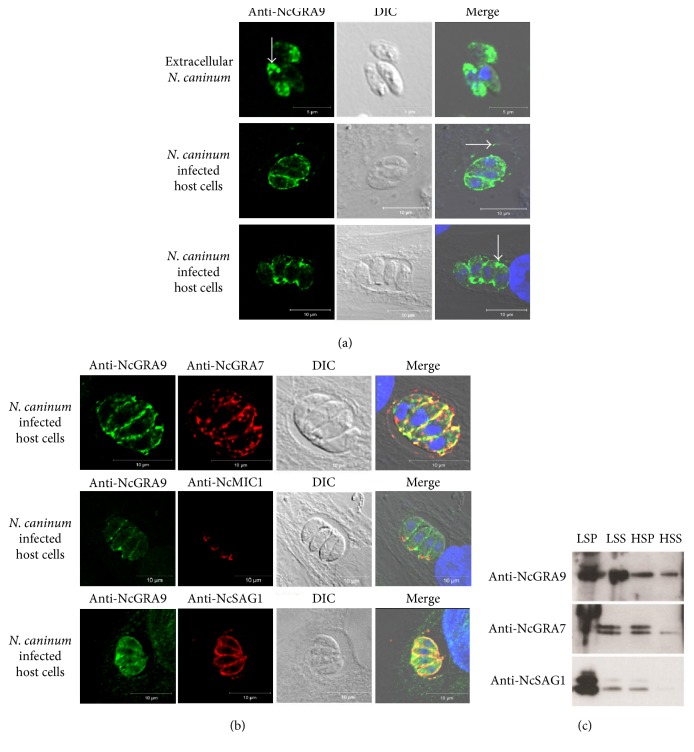
In (a) the localization of NcGRA9 was analysed by confocal microscopy to show that it belongs to the dense granule proteins of* N. caninum*. In extracellular tachyzoites NcGRA9 was detectable in a dotted pattern (white arrow, top panel) implying its localization in dense granules. In* N. caninum* infected host cells NcGRA9 was secreted into the PV. The protein localized either near the PVM (white arrow, middle panel) or in the vacuolar space near the tachyzoites (white arrow, lowest panel). (b) shows colocalization studies of NcGRA9 with NcGRA7 (PV/PVM marker), NcMIC1 (apical marker), and NcSAG1 (cell surface marker). NcGRA9 colocalizes partially with NcGRA7 and NcSAG1, but not with NcMIC1. In (c) cell fractionation by ultracentrifugation was performed with* N. caninum* infected HFF. It shows the targeting behaviour of NcGRA9 after secretion into the host cell. NcGRA9 was present in the low speed pellet (LSP) which includes host cell debris and intact parasites, but also in the low speed supernatant (LSS) which was further separated into a membranous fraction (high speed pellet, HSP) and a soluble fraction (high speed supernatant, HSS) by ultracentrifugation. Secreted NcGRA9 is membrane-bound (HSP) as well as soluble (HSS) in the vacuolar space. The control protein NcGRA7 behaves as TgGRA7 and is mainly membrane-bound after secretion. The surface antigen NcSAG1 is mainly detectable in the LSP fraction which contains the intact parasites, as expected. Green: anti-NcGRA9 cy2; blue: DAPI; merge: anti-NcGRA9, DAPI, DIC (differential interference contrast).

In order to perform localization studies, two different polyclonal antisera against recombinant NcGRA9 were produced in rabbits. These antisera were used to localize NcGRA9 within* N. caninum* tachyzoites by immunofluorescence. In extracellular parasites, NcGRA9 was found in a dotted pattern distributed all over the tachyzoite, which is a staining pattern reminiscent for dense granule organelles (Figure 4(a)). This implies that the protein is located inside the dense granules which are secretory organelles of* N. caninum*. Following invasion, NcGRA9 is found within the lumen of the PV and is usually detectable in the vacuolar space next to the tachyzoites and near the periphery of the PV, possibly associated with the PV membrane (PVM) (Figure 4(a)). Further colocalization analyses revealed that NcGRA9 colocalizes with NcGRA7 in the vacuolar space next to the tachyzoites but not to NcGRA7 localized to the near periphery of the PV (Figure 4(b)). Additionally NcGRA9 did not colocalize with NcMIC1 at the apical complex (Figure 4(b)). Overlay of NcSAG1, a tachyzoite surface protein, with NcGRA9 revealed a partial colocalization nearby the parasite membrane (Figure 4(b)), which might be explained by the close proximity of the extracellular NcGRA9 signal and the surface-bound NcSAG1 labeling.

To investigate the targeting behaviour of NcGRA9 within the PV in more detail, cell fractionation analysis by ultracentrifugation was carried out (Figure 4(c)). As expected NcGRA9 was observed in the low speed pellet (LSP) fraction which contains intact tachyzoites. The low speed supernatant (LSS) was further separated by high speed centrifugation, yielding a high speed pellet (HSP) and respective supernatant (HSS). Western blotting revealed that NcGRA9 is present in both fractions. This indicates that NcGRA9 might be physically associated with membranes or protein complexes (shown by the presence in HSP) and also as a soluble protein (as indicated in HSS) in the vacuolar space. As a control, the distribution of NcGRA7, a membrane-bound GRA protein, was analysed. We found that NcGRA7 was present in the LSP, LSS, and HSP. The* T. gondii* homologue TgGRA7 was found to be associated with membranes when secreted into the PV [18]. Thus, the distribution of NcGRA7 in nonsoluble fractions is in accordance with what has been found for TgGRA7. In contrast, the major surface protein NcSAG1, which is known not to be secreted, was only detectable in the LSP, whereas degradation products were present only in very low amounts in LSS and HSP.

**Figure 5:**
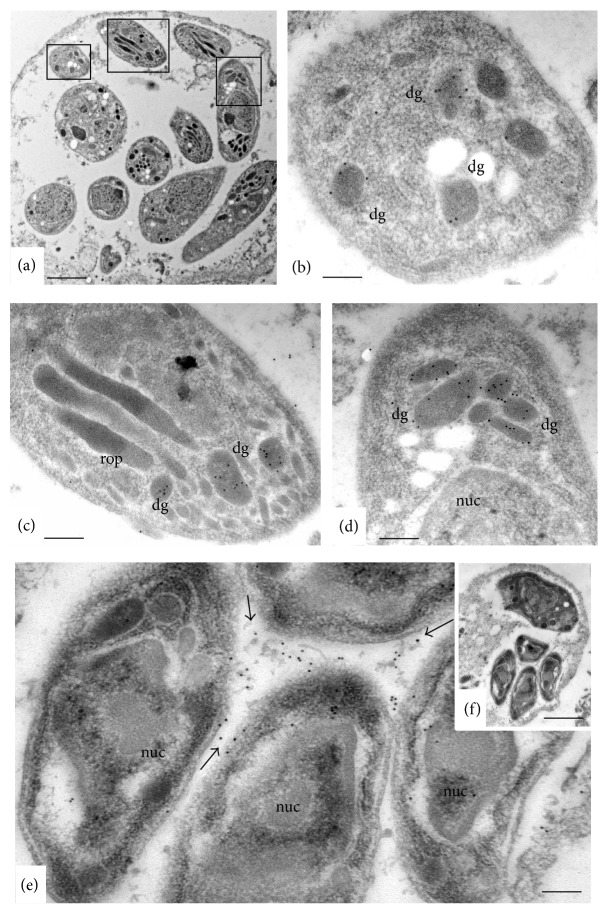
(a) shows a low magnification view of LR-White embedded* N. caninum* tachyzoites forming an intracellular PV in HFF. The boxed areas in (a) are shown at higher magnification in (b), (c), and (d). dg = dense granules, rop = rhoptries, mic = micronemes, and nuc = nucleus. (e) and (f) show another PV, where the intravacuolar membranous network is stained with anti-NcGRA9 antibodies (arrows). (e) shows the boxed area in (f) at higher magnification. Bars in (a) = 1 *μ*m, (b) = 0.14 *μ*m, (c) and (d) = 0.2 *μ*m, (e) = 1.8 *μ*m, and (f) = 1.6 *μ*m.

The localization of NcGRA9 was also studied by immunogold TEM. NcGRA9 labeling was evident in the parasite dense granules, but no gold particles could be seen within the tachyzoite nuclei, rhoptries, and micronemes (Figures 5(a)–5(d)). In many instances, NcGRA9 labeling was found to be associated with the PV network (Figures 5(e) and 5(f)).

**Figure 6:**
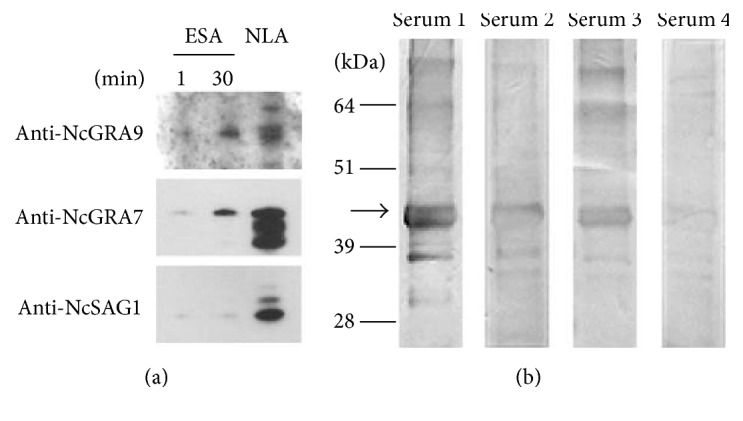
(a) shows that NcGRA9 belongs to the “excreted secreted antigens” (ESA) of* N. caninum*. The amount of NcGRA9 and the NcGRA7 control protein accumulated over time. In contrast NcSAG1 control protein was not present in ESA. In (b) sera from* N. caninum* infected cattle (diluted 1 : 50) were tested for their recognition of recombinant NcGRA9 protein (5 *μ*g) via Western Blot analyses. Three out of four cattle sera contained antibodies against recombinant NcGRA9 (black arrows). NLA: Neospora lysate antigen.

GRA proteins are constitutively secreted and therefore belong to the group of “excreted secreted antigens” (ESAs), some of which are immunodominant antigens [6]. Western blotting of* N. caninum* ESA showed that NcGRA9 is indeed secreted (Figure 6(a)). The amount of secreted NcGRA9 increased over time (between 1 to 30 min) in cell-free culture medium containing 10% FCS. As expected, NcGRA7 was also detected in increasing amounts in cell supernatants. In contrast, NcSAG1 content in ESA did not increase over time, indicating that the tachyzoites had remained intact during the incubation period.

As GRA proteins have been recognized as immunodominant antigens, sera from chronically infected cattle were analysed for the presence of anti-NcGRA9 antibodies by Western blotting.  Figure 6(b) shows that three of four sera obtained from* N. caninum* infected cattle reacted positively with recombinant NcGRA9.

### 3.3. Analysis of Transfected Full-Length and C-Terminally Truncated NcGRA9

**Figure 7:**
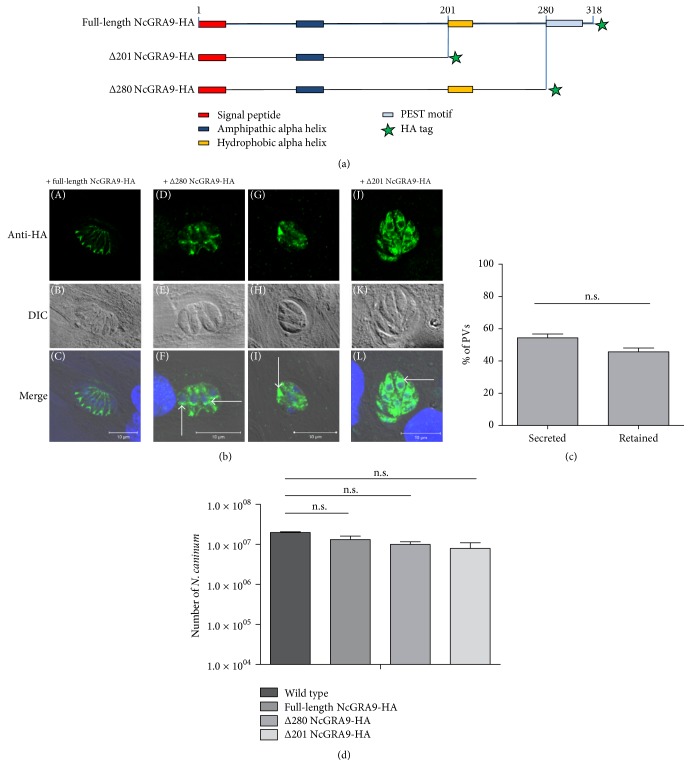
(a) shows that the full-length sequence of NcGRA9 contains an N-terminal signal peptide (red), an alpha helix with amphipathic characteristics (blue), a hydrophobic alpha helix (yellow), and a PEST motif at the C-terminus of the protein (light blue). An HA-tagged version of the full-length protein and two C-terminal truncations (Δ201, Δ280) were stably expressed in* N. caninum* tachyzoites. In (b) the localization was analysed by confocal microscopy. Full-length NcGRA9-HA was secreted into the PV and is located near the PVM or in the vacuolar space near the tachyzoites (A, B). Δ280 NcGRA9-HA either retained inside the tachyzoites especially anterior of the nucleus (C, D, white arrows) or was secreted into the vacuolar space (E, F, white arrow). Δ201 NcGRA9-HA always retained inside the tachyzoites in the paranuclear space (G, H, white arrow). (c) shows the enumeration of Δ280 NcGRA9-HA localization in 100 PV in three independent immunofluorescence experiments. No significant differences were observed (n.s. = not significant). In (d)* N. caninum* tachyzoite growth upon additional expression of NcGRA9 variants in HFF was analysed. Therefore, quantitative real-time PCR was performed. No significant differences in parasite numbers were observed (n.s. = not significant). Green: anti-HA cy2; blue: DAPI; merge: anti-HA, DAPI, DIC (differential interference contrast).

In silico analysis of the protein sequence revealed that NcGRA9 exhibits a N-terminal, hydrophobic signal peptide, while the bulk of the protein, with few exceptional sequences, is rather hydrophilic and contains several alpha helices (Figure 3(a)). To analyse the impact of these alpha helices on the targeting behaviour of NcGRA9, full-length NcGRA9 and two C-terminally truncated proteins containing a C-terminal HA-tag were generated (Figure 7(a)). The two truncated versions Δ280 NcGRA9-HA and Δ201 NcGRA9-HA exhibit altered C-terminal domains, ending just before the PEST motif (amino acid 280), and just prior to a hydrophobic alpha helix (amino acid 280), respectively.

The different variants of NcGRA9-HA were cloned into the vector system pDHFR-TSc3 [10],* N. caninum* was transfected by electroporation, and then parasites were grown under pyrimethamine selection pressure. After establishing stable clonal lines, the localization of full-length NcGRA9-HA, Δ280 NcGRA9-HA, and Δ201 NcGRA9-HA was monitored in* N. caninum* infected HFF by immunofluorescence at 24 h postinfection (Figure 7(b)). Full-length NcGRA9-HA was secreted into the PV space and showed a localization similar to endogenous NcGRA9. Δ280 NcGRA9-HA did not show the same localization, and its distribution was inconsistent. In some PVs, Δ280 NcGRA9-HA was found exclusively within the parasites, while in other PVs Δ280 NcGRA9-HA was detectable also in the vacuolar space (Figure 7(b)). The distribution of the truncated protein was analysed in 100 PVs per experiment (three independent experiments). In 54% of all PVs Δ280 NcGRA9-HA was secreted into the PV whereas in 46% of all PVs Δ280 NcGRA9-HA remained inside the parasites and no significant difference was observed (Figure 7(c)). In contrast, the localization of Δ201 NcGRA9-HA was inside* N. caninum* tachyzoites and this variant was never found to be secreted into the vacuolar space (Figure 7(b)).

To investigate the impact of these heterologously expressed proteins on the viability of the tachyzoites, proliferation assays were performed. There were no significant differences in growth behaviour between the different NcGRA9 versions compared to endogenous NcGRA9 (Figure 7(d)).

**Figure 8:**
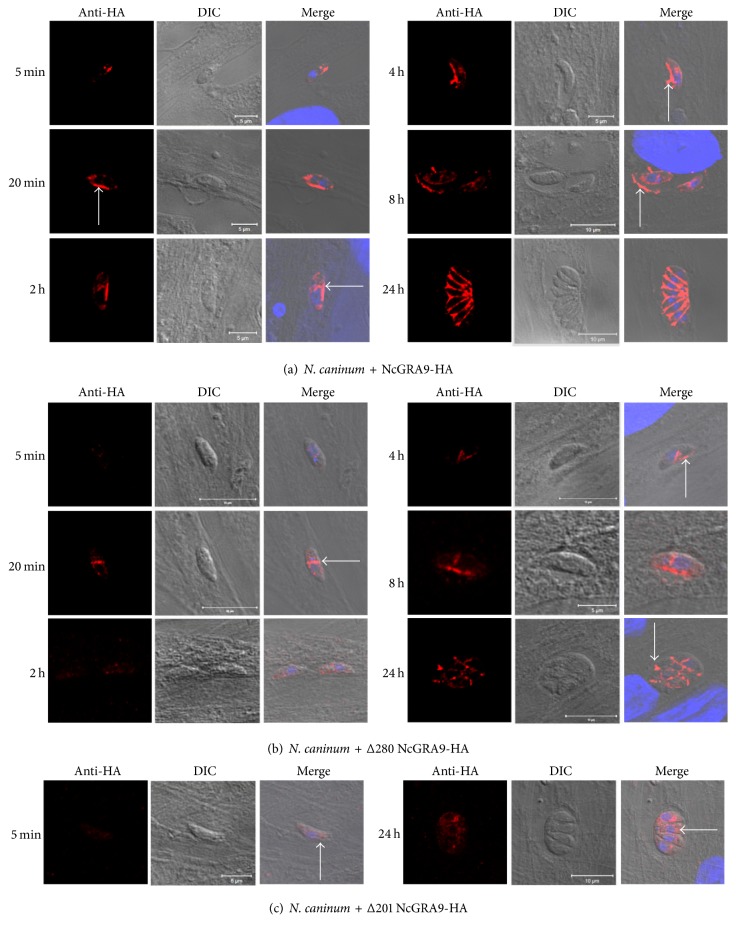
The secretion of the HA-tagged NcGRA9 variants was analysed over time (5 min, 20 min, 2 h, 4 h, 8 h, and 24 h postinfection) by confocal microscopy. (a) shows that full-length NcGRA9-HA was present in the vacuolar space after 20 min (white arrow). After 4 h the protein was in the deeper vacuolar space (white arrow) and after 8 h full-length NcGRA9-HA was also present near the PVM (white arrow). (b) shows that Δ280 NcGRA9-HA secretion is delayed compared to full-length NcGRA9-HA and starts 4 h after invasion (white arrow). (c) shows that Δ201 NcGRA9-HA was retained at all analysed time points inside the tachyzoites in the paranuclear space (white arrows).Red: anti-HA cy3; blue: DAPI; merge: anti-HA, DAPI, DIC.

In addition, we analysed the secretion kinetics of the transfected NcGRA9-HA variants at different time points (5 min to 24 h) after invasion. As can be seen in Figure 8(a), full-length NcGRA9-HA was detected within the vacuolar space at 20 min postinfection, and at 8 h postinfection, the protein accumulated near the PV membrane. In comparison, secretion of Δ280 NcGRA9-HA was delayed (Figure 8(b)), and this truncated NcGRA9 variant was found within the PV only after 4 h postinvasion. Δ201 NcGRA9-HA was not secreted into the PV and retained inside the parasites for the whole duration of the experiment (Figure 8(c)).

## 4. Discussion

We here report on the characterization of the dense granule protein NcGRA9 of the apicomplexan parasite* N. caninum*. NcGRA9 is encoded by a single-copy gene located on chromosome XII of the* N. caninum* genome and contains two exons and one intron. These findings correlate with the description of the* Tggra9* gene [19] of the closely related* T. gondii*.

The deduced NcGRA9 protein sequence was studied in silico with regard to alpha helical regions, hydrophilicity, and amphiphilic helices. Overall, NcGRA9 is a mainly hydrophilic protein but also contains shorter hydrophobic regions. We identified several alpha helical regions, three of which exhibit amphiphilic properties with potential functions in protein-protein interactions [6]. The putative N-terminal signal peptide, which targets the protein into the secretory pathway, is comprised of 19 amino acids. N-terminal signal peptides are a hallmark of secretory proteins and have been described in all GRA proteins of* T. gondii* and* N. caninum* identified so far [6]. Alignment of NcGRA9 and TgGRA9 protein sequences resulted in a high sequence identity of 60%, and TgGRA9 also exhibited similar hydrophilicity, hydrophobic domains, and alpha helices. Other* T. gondii* and* N. caninum* GRA homologues are less conserved. For instance, GRA1 of* T. gondii* and* N. caninum* have only 33.5% identical amino acid sequences [20]; GRA2 exhibits an identity of 52% [21], GRA6 of 33.5% [22], GRA7 of 34% [23], and MAG1 of 53% [24]. Thus, the high level of GRA9 sequence conservation could indicate that this protein is involved in a functional activity that is important, and probably similar, in both species.

Polyclonal antisera raised against recombinant NcGRA9 reacted with a protein band with a relative molecular mass of 39 kDa. This is notably higher than the theoretical molecular mass of NcGRA9, which based on its amino acid sequence would be 34.9 kDa. Similar discrepancies between theoretical and observed molecular weight have been described for other GRA proteins. Such differences could arise due to posttranslational modifications, which could alter the electrophoretic mobility of proteins, or due to proline-rich sequences, as described for TgGRA8 [25]. The NcGRA9 proline content is 8.2%. Such an accumulation of proline in the primary sequence can be responsible for reduced electrophoretic mobility depending on kinks and structural rigidity [26].

Immunofluorescence localization studies showed that NcGRA9 was present in a dotted pattern, distributed all over the parasite. Such a pattern is typical for GRA proteins, as shown by others [27–29], and implies that NcGRA9 is indeed localized within the dense granules. After invasion into the host cells, NcGRA9 was secreted into the PV and either remained in the vacuolar space in the vicinity of the tachyzoites or localized to the PV periphery. Secretion into the PV following invasion is a characteristic trait of GRA proteins [6], some of which associate with the membranous tubular network that forms the vacuolar matrix such as NcMAG1 [24] or associate with the PVM, for example, NcGRA7 [27]. Immunogold-EM confirmed the localization of NcGRA9 in the parasite dense granules and also showed that intravacuolar NcGRA9 was associated with the PV membranous tubular network. An association of NcGRA9 with the PV membrane was not detected; however, this membrane is admittedly not very well conserved in LR-White embedded specimens [27–29], and thus it is not possible to make a definitive statement on that particular localization.

This fits the results of our biochemical fractionation of soluble and membrane- or protein aggregate-associated proteins of the PV content by ultracentrifugation. It showed that NcGRA9 was present as a soluble protein within the vacuolar space (as indicated by the presence of this protein in the high speed supernatant), and NcGRA9 was also found in the high speed pellet which contains the membranous fraction. This is in accordance with our previous findings on TgGRA9, which was shown to associate with the PVM and the membranous tubular network in the PV [11]. Therefore, it is conceivable to conclude that after secretion NcGRA9 is directly or indirectly bound to membranes. NcGRA7, previously shown to be associated with the tubular network of the PV matrix and the PVM by immunogold-electron microscopy [27], was mainly present in the membrane (high speed pellet) fraction, and similar findings had been obtained on TgGRA7 [30]. In contrast to these GRA proteins, the major tachyzoite surface antigen NcSAG1 remained tightly associated with the parasites and was found only in the low speed pellet, and this coincides with earlier studies [31, 32].

NcGRA9 is also a prominent component of the ESA fraction of* N. caninum.* ESA were first described in* T. gondii* and recognized as immunodominant antigens [33]. Recently, Pollo-Oliveira et al. [8] analysed the secreted fraction of* N. caninum* using mass spectrometry and identified a TgGRA9-like protein as one of the most abundant proteins. Our analysis of ESA by Western Blot with an NcGRA9-specific antiserum confirmed that NcGRA9 is a secreted protein. In addition, three of four sera obtained from cattle chronically infected with* N. caninum* reacted with recombinant NcGRA9, indicating a robust humoral immune response against this antigen. A recent study has shown that sera derived from* T. gondii*-infected turkeys and chicken contained antibodies against TgGRA9, and the magnitude of the antibody response depended on the infection mode (tachyzoites versus oocysts) and on the time point of sample collection [34]. An elaborated work that comprehended several TgGRA single and double knockouts revealed that TgGRA proteins are important for the formation of the intravacuolar network and the replication rates* in vitro* [35], indicating the importance of the TgGRA proteins for the parasite. That is further confirmed by the fact that some TgGRA proteins (TgGRA15, TgGRA16, and TgGRA24) are targeted to the host cell nucleus, where they modulate the host cell response [36].

It is described that similar antimicrobial effector mechanisms are active against* N. caninum* and* T. gondii*. We found that the induction of the tryptophan-degrading enzyme indoleamine 2,3-dioxygenase by interferon-gamma is responsible for the antiparasitic effect against* T. gondii* and* N. caninum* in human and bovine fibroblasts [37], while immunity related GTPases and guanylate-binding proteins mediate the inhibition of both parasites in cells of murine origin [38].

While the hydrophobic signal peptide is targeting NcGRA9 into the secretory pathway, we aimed to define protein regions which were important for the actual secretion process of NcGRA9. Thus we expressed three different HA-tagged NcGRA9 variants in* N. caninum* tachyzoites and analysed secretion at different time points postinvasion. The expression of additional recombinant variants of the NcGRA9 protein did not alter tachyzoite viability or proliferation kinetics. The secretion pattern of recombinant full-length NcGRA9-HA was identical to endogenous NcGRA9. In contrast, the C-terminally truncated Δ280 NcGRA9-HA, lacking the PEST domain, either remained within the parasite or exhibited delayed secretion. Moreover, Δ201 NcGRA9-HA, lacking the PEST domain and a hydrophobic alpha helix, was not secreted at all.

In addition, we analysed the localization of the HA-tagged recombinant proteins within the intravacuolar tachyzoites. At 24 h postinvasion, full-length NcGRA9-HA was not detectable anymore within the intravacuolar parasites, since it was completely secreted into the PV. In contrast, in 46% of all Δ280 NcGRA9-HA-expressing tachyzoites, the HA-tagged protein was retained inside the parasite anterior to the nucleus. This localization indicates that Δ280 NcGRA9-HA is retained in the Golgi apparatus [39]. Thus, the C-terminal PEST domain could facilitate the transport from the Golgi apparatus to the dense granules. Δ201 NcGRA9-HA, missing the PEST domain and a hydrophobic alpha helix, was never found in the vacuolar space but was detectable in the paranuclear region of the intravacuolar tachyzoites. Thus, this protein could be localized in the endoplasmic reticulum (ER) [39]. It has been shown that improperly folded proteins could be retained in Golgi complex or ER by chaperone complexes, and this is a well-documented quality control step in eukaryotic cells [40]. Striepen et al. [41] described retention of MIC3 proteins in the region of* T. gondii* ER or Golgi apparatus after truncation of the cysteine-rich central portion. According to these data we suggest that C-terminally truncated NcGRA9 proteins were retained in these organelles due to improper folding and might be subsequently degraded.

In summary, our data show that NcGRA9 is an immunodominant* N. caninum* dense granule protein with high similarities to TgGRA9. The C-terminal PEST domain of NcGRA9 is important for the correct folding and targeting to the dense granules, and subsequent secretion into the PV is at least partially mediated by the C-terminal hydrophobic alpha helix and the PEST domain. The function of NcGRA9 and the role of this protein in the life cycle of* N. caninum* are not known, and in future experiments NcGRA9-deficient parasites could be supplemented with different variants of the* gra9* gene to define the functional activity of this protein.

## Figures and Tables

**Table 1:** Oligonucleotides used in this study.

1-1697for	5′-CACCACATGATGAGGTCATTCAAG-3′
1-1697rev	5′-CACCACTTATATTTCTCCGTTATGGTT-3′
271-1290for	5′-CACCACGTTCAGGAGATGATGCGG-3′
271-1290rev	5′-CACCACGACTCTCCCCGTGCT-3′
probeNcGRA9for	5′-CGTGCGCGTGGTGTTGCACTT-3′
probeNcGRA9rev	5′-TATTTCTCCGTTATGGTTCGAGAAACG-3′
NcGRA9NdeIfor	5′-CACCACCATATGATGATGAGGTCATTCAAG-3′
NcGRA9HindIIIre	5′-ATAATAAAGCTTTATTTCTCCGTTATGGTTCGAGAA-3′
pDHFRSpeINotIfo	5′-CACCACACTAGTAAGCTTCGCGAGGCTGTAAATCCCGTG-3′
pDHFRSpeINotIre	5′-ATAATAGCGGCCGCCCATGGCCTAGGCTTCCCGGAT-3′
GRA9HAAvrNcofor	5′-CACCACCCTAGGATGATGAGGTCATTCAAGTCC-3′
GRA9HAAvrNcorev	5′-ATAATACCATGGTTAAGCGTAATCTGGAACATCGTATGGGTATATTTCTCCGTT-3′
3′GRA9NcoINotIf	5′-CACCACCCATGGGCGCCACGTGTA-3′
3′GRA9NcoINotIr	5′-ATAATAGCGGCCGCTTCGGAACACACTACG-3′
NcGRA9d280revHA	5′-ATAATACCATGGTTAAGCGTAATCTGGAACATCGTATGGGTACTCTGTAGGAGT-3′
NcGRA9d201revHA	5′-ATAATACCATGGTTAAGCGTAATCTGGAACATCGTATGGGTAGCACTCAGGGTC-3′
Nc5forqPCR	5′-CGAGAGTTCAGTGTTCTGTGTTGA-3′
Nc5revqPCR	5′-TCGTCCGCTTGCTCCCTAT-3′
Nc5 Probe	FAM-CAACACCGGCGGCACTGATGA-BHQ1
